# Comparative chromosome painting in hummingbirds (Trochilidae)

**DOI:** 10.1590/1678-4685-GMB-2020-0162

**Published:** 2020-11-30

**Authors:** Tiago Marafiga Degrandi, Ivanete de Oliveira Furo, Edivaldo Herculano Correia de Oliveira, Alice Lemos Costa, Malcolm A. Ferguson-Smith, Patrícia C.M O’Brien, Jorge C. Pereira, Analía Del Valle Garnero, Ricardo José Gunski, Roberto Ferreira Artoni

**Affiliations:** 1 Universidade Estadual de Ponta Grossa (UEPG), Programa de Pós-Graduação em Biologia Evolutiva, Ponta Grossa, PR, Brazil; 2 Laboratório de Reprodução Animal, LABRAC, Universidade Federal Rural da Amazônia, UFRA, Parauapebas, PA, Brazil.; 3 Universidade Federal do Pará (UFPA), Programa de Pós-Graduação em Genética e Biologia Molecular, Belém, PA, Brazil.; 4 Instituto Evandro Chagas (IEC), Ananindeua, PA, Brazil.; 5 University of Cambridge, Cambridge Resource Centre for Comparative Genomics, Department of Veterinary Medicine, Cambridge, UK.; 6 Animal and Veterinary Research Centre (CECAV), University of Trás‐os‐Montes and Alto Douro (UTAD), Vila Real, Portugal.; 7 Universidade Federal do Pampa (UNIPAMPA), Programa de Pós-Graduação em Ciências Biológicas, São Gabriel, RS, Brazil.

**Keywords:** Karyotype, FISH, bird, chromosome, evolution

## Abstract

Hummingbirds (Trochilidae) are one of the most enigmatic avian groups, and also among the most diverse, with approximately 360 recognized species in 106 genera, of which 43 are monotypic. This fact has generated considerable interest in the evolutionary biology of the hummingbirds, which is reflected in a number of DNA-based studies. However, only a few of them explored chromosomal data. Given this, the present study provides an analysis of the karyotypes of three species of Neotropical hummingbirds, *Anthracothorax nigricollis* (ANI), *Campylopterus largipennis* (CLA), and *Hylocharis chrysura* (HCH), in order to analyze the chromosomal processes associated with the evolution of the Trochilidae. The diploid number of ANI is 2n=80 chromosomes, while CLA and HCH have identical karyotypes, with 2n=78. Chromosome painting with *Gallus gallus* probes (GGA1-12) shows that the hummingbirds have a karyotype close to the proposed ancestral bird karyotype. Despite this, an informative rearrangement was detected: an in-tandem fusion between GGA7 and GGA9 found in CLA and HCH, but absent in ANI. A comparative analysis with the tree of life of the hummingbirds indicated that this fusion must have arisen following the divergence of a number of hummingbird species.

## Introduction

Hummingbirds (family Trochilidae) form one of the most enigmatic and diverse avian groups, with some 360 recognized species representing 106 genera, of which, 43 are monotypic (Gill and Donsker, 2018). These birds are exclusive to the New World, although fossils from the early Oligocene indicate that they may have originated in Europe around 34-28 million years ago, and subsequently dispersed to South America through Beringia (Mayr, 2004, 2007; Bochenski and Bochenski, 2008; Mcguire *et al*., 2014). In the New World, these birds have established intimate evolutionary relationships with a wide range of angiosperms through adaptations for nectar feeding. These adaptations have allowed the hummingbirds to occupy an enormous range of ecological niches within their geographic range, which extends from Alaska and Canada to Tierra del Fuego, in Argentina (Feinsinger and Colwell, 1978). 

The family Trochilidae has been the subject of a number of DNA studies (Bleiweiss *et al*., 1997; Bleiweiss, 1998; Graham *et al*., 2009; Mcguire *et al*., 2007, 2008, 2014). Using a multilocus DNA approach, McGuire *et al*. (2014) concluded that the considerable diversity of trochilid species was the result of a rapid evolutionary radiation, which occurred 22 million years ago. These authors defined nine hummingbird clades: Bees, Brilliants, Coquettes, Emeralds, Hermits, Mangoes, Mountain Gems, *Patagona*, and Topazes. Trochilids have also been the subject of considerable taxonomic controversy, being originally assigned to order Apodiformes (Apodidae, Hemiprocnidae, and Trochilidae), and were later elevated to their own order, the Trochilifomes, which included only the family Trochilidae (Sibley and Ahlquist, 1990). More recent analyses of the complete bird genome have nevertheless assigned the hummingbirds to the order Caprimulgiformes, which also includes the Apodidae and the nightjars, family Caprimulgidae (Jarvis *et al*., 2014).

Despite the considerable interest in the evolutionary biology of the hummingbirds, very few cytogenetic data are available, and little is known of the chromosomal complement of these diminutive birds. *Calypte anna* was the first species to be karyotyped (Beçak *et al*., 1973), with 2n=74; afterwards, four species - *Amazilia lactea*, *Colibri serrirostris*, *Lophornis magnificus*, and *Chlorestes notatus* - were analyzed and showed the same diploid number (2n=82) (Christidis, 1990). 

The study of bird karyotypes and chromosome structure has helped to elucidate important evolutionary questions, in particular through the identification of phylogenetically informative chromosomal signatures (Griffin *et al*., 2007; Kretschmer *et al*., 2018, Degrandi *et al*., 2020a). The advances obtained by Fluorescence *in situ* Hybridization (FISH) analyses using whole chromosome probes (WCP) of *Gallus gallus* 2n=78 (GGA 1-10) have shown that the macrochromosomes are conserved completely among highly divergent lineages from the Paleognathae to the Neognathae groups (Griffin et al., 1999; de Oliveira *et al*., 2005; Nishida-Umehara *et al*., 2007; Kretschmer *et al*., 2014). 

Despite the value of cytogenetic data for evolutionary analyses, less than 10% of all birds have been karyotyped (Degrandi *et al*., 2020b). This lacuna is even larger for chromosome painting, which has been applied to less than 1% of bird species, and in fact, many bird orders and families, including the Trochilidae, lack any data concerning comparative chromosome painting (Degrandi *et al*., 2020b). Given this, the present study investigated the evolutionary processes that have molded the chromosomal characteristics of the trochilids, from the perspective of their karyotype evolution and their phylogenetic relationships with other birds.

## Material and Methods

Samples of three hummingbird species - *Anthracothorax nigricollis* (ANI), *Campylopterus largipennis* (CLA), and *Hylocharis chrysura* (HCH) - were collected during field expeditions in Porto Vera Cruz, in the state of Rio Grande do Sul, Brazil, and in Belém, in the Brazilian state of Pará. A single female of each species was captured, according to the norms established by federal specimen collecting license SISBIO number 61047-2 and the Research Ethics Committee (UNIPAMPA 010/2018). 

Mitotic chromosomes were obtained from a culture of fibroblasts, following Sasaki et al. (1968), with modifications. In brief, skin biopsies were collected and cells were dissociated in Colagenase type IV solution (0.45%) at 37 °C for 1 h. Cell suspensions were then added to 25 cm^2^ culture flasks containing 5 mL of DMEM (GIBCO) medium supplemented with 20% fetal bovine serum, 100 u/ml of penicillin, and 100 μg/ml of streptomycin, and incubated at 37 ^o^C. Cell growth was monitored daily and, when satisfactory, cell division was blocked by the addition of 100 µl of 0.005% Colchicine directly into the flask, which was then incubated at 37 ºC for 4 h. Subsequently, there followed hypotonic treatment with KCl solution (0.75 M) for 20 minutes, and fixation by three washes with methanol and acetic acid (3:1).

For each species, the diploid number was established by the analysis of 40 metaphases stained with Giemsa under an optical microscope, with a 100 x lens. The complete karyotype of each species was organized and the chromosome morphology classes were determined using the centromeric index (CI), following Guerra (1986). 

Whole chromosome probes of *G. gallus* (GGA), covering the first 12 pairs (Cambridge Resource Center for Comparative Genomics, Cambridge, UK) were used in comparative chromosome painting. The probes were labeled by DOP-PCR, with biotin or digoxigenin, and detected using streptavidin-CY3 and/or anti-digoxygenin-fluorescein (Telenius *et al*., 1992). FISH experiments followed de Oliveira et al. (2010). The results of the FISH-WCP procedure were analyzed and photographed under a Zeiss microscope with a 63 x lens and Axiovision 4.8 software (Zeiss, Germany).

## Results

**Figure 1 -:**
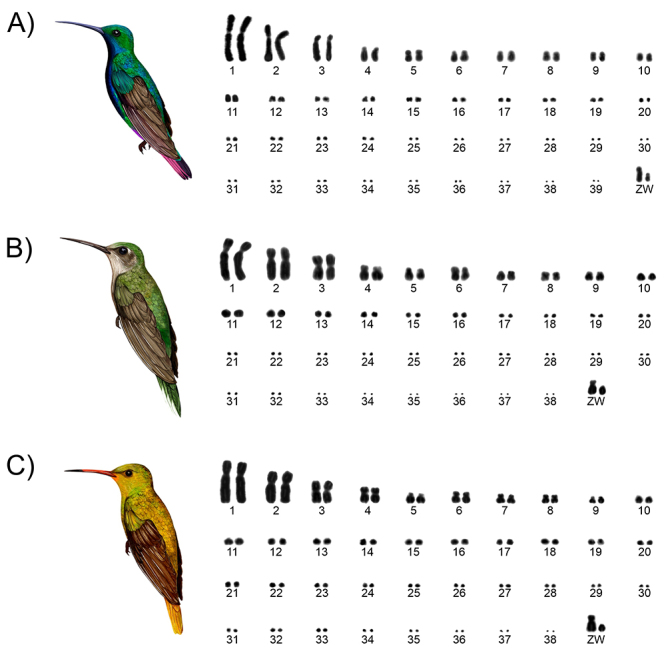
Karyotypes of the three hummingbird species (family Trochilidae) analyzed in the present study. (A) *Anthracothorax nigricollis* 2n=80, (B) *Campylopterus largipennis* 2n=78, and (C) *Hylocharis chrysura* 2n=78.

The diploid number of *A. nigricollis* is 2n=80 chromosomes (Figure 1A). The macrochromosomes (1, 2, 6, 7, 8, 9, Z and W) are submetacentric, while 5 is metacentric, 3 and 4 are acrocentric, and chromosomes 10 through 39 are all telocentric, forming a gradual decline in the length of the chromosomes. Identical karyotypes of 2n=78 chromosomes were observed in *C. largipennis* (Figure 1B) and *H. chrysura* (Figure 1C). In both cases, macrochromosomes 1, 2, 4, 6, 7, 8, 9, and Z, are submetacentric, 3 is metacentric, 5 is acrocentric, and chromosomes 10 through 38, plus the W are all telocentric.

**Figure 2 -:**
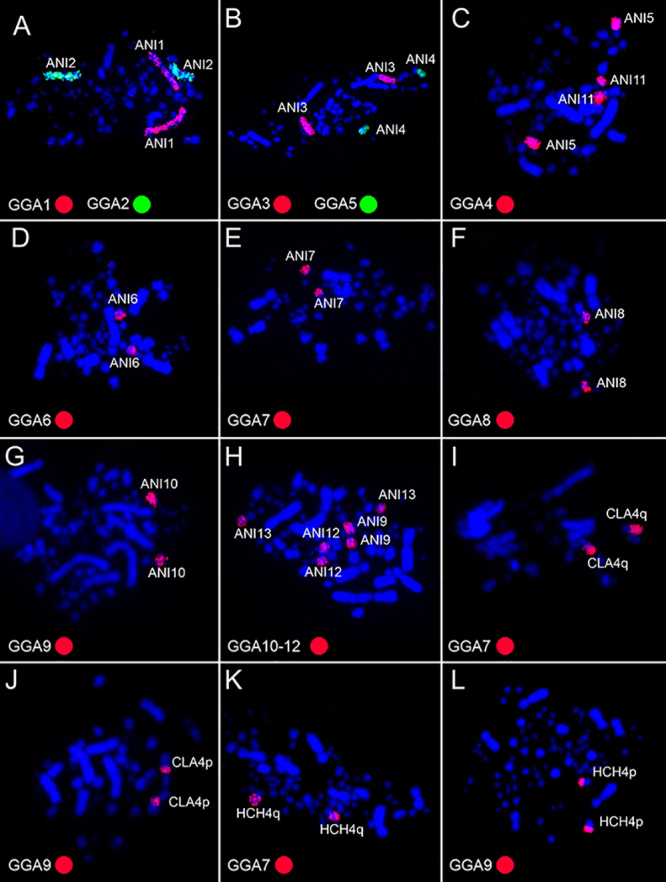
Representative metaphases showing Fluorescence *in situ* Hybridization using *Gallus gallus* (GGA 1-GGA12) chromosomal probes in *Anthracothorax nigricollis*, ANI (metaphases A-H), and the GGA7 and GGA9 probes in *Campylopterus largipennis*, CLA (metaphases I and J) and *Hylocharis chrysura*, HCH (metaphases K and L).

**Figure 3 -:**
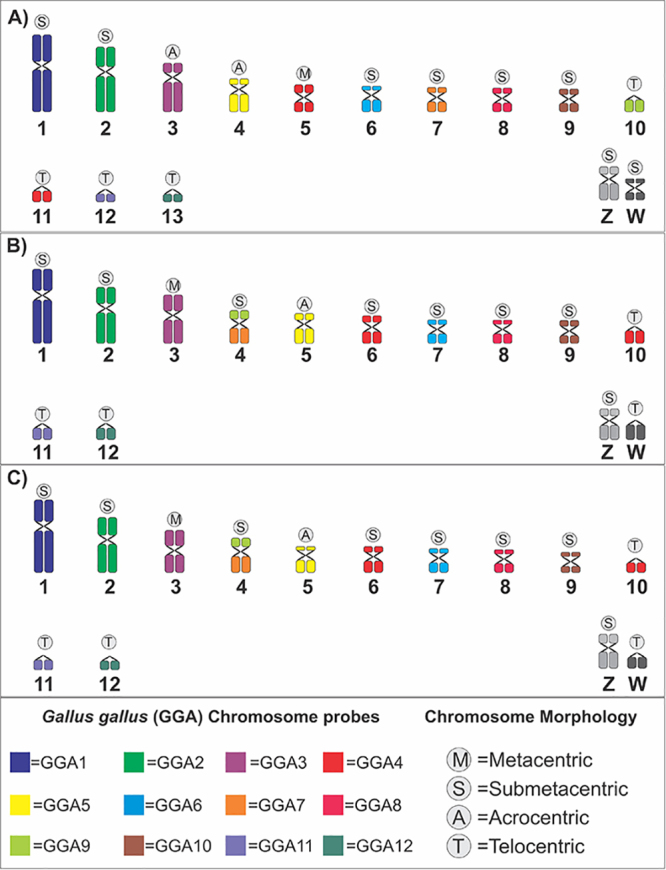
Comparative ideograms showing the homologies among the macrochromosomes of the hummingbirds *Anthracothorax nigricollis* (A), *Campylopterus largipennis* (B), and *Hylocharis chrysura* (C). This scheme was obtained by Fluorescence *in situ* Hybridization using the *Gallus gallus* chromosomal probes (GGA 1-GGA12).

The comparative chromosome painting indicated that the syntenies corresponding to GGA1-GGA12 were conserved in *A. nigricollis*, with the exception of GGA4, which corresponded to two distinct pairs (Figure 2 A-H). Similar results were found in *C. largipennis* and *H. chrysura,* except for pairs GGA7 and GGA9, which were fused in a single chromosome pair, corresponding to chromosomes 4q (GGA7) and 4p (GGA9) in the two species (Figure 2 I-L). The chromosomal homology between the three species was represented in ideograms and is shown in Figure 3.

## Discussion

Hummingbirds show karyotypes similar to those found in the majority of birds, with diploid numbers of around 2n=80, together with the preservation of the syntenies corresponding to *G. gallus* (GGA) macrochromosomes. This uniformity of bird karyotypes has been known since the first cytogenetic studies in these animals, which reported only basic chromosome numbers and the structural characteristics of the karyotype (Ohno *et al*., 1964; Garnero *et al*., 2006). These observations were confirmed subsequently by chromosome painting, which supports that the putative ancestral karyotype of the birds (PAK) had 2n = 80 chromosomes (Griffin *et al*., 2007).

In this work, although it included a small number of species in the analysis, the homology maps (Figure 3) compared to *G. gallus* reveal that *A. nigricollis*, *C. largipennis* and *H. chrysura* show highly similar karyotypes (Figure 1), which preserve most of the syntenic groups represented by the *G. gallus* macrochromosome probes (GGA1, GGA2, GGA3, GGA5, GGA6, GGA8, GGA10, GGA11, and GGA12) (Figures 2 and 3). However, it was possible to observe that the karyotypes of *C. largipennis* and *H. chrysura* correspond to one another in chromosome number and morphology, and differ from the karyotype of *A. nigricollis*, by a centric fusion between chromosomes homologous to GGA7 and GGA9. 

These findings are consistent with the most recent phylogeny of the hummingbirds, in which *A. nigricollis* is included in a separate clade, while the other two species are sister groups. Hence, *A. nigricollis* was assigned to the Mangoes clade, one of the first that diverged 20 million years ago (Ma), while *C. largipennis* and *H. chrysura* belong to the Emeralds clade, which arose about 8 Ma later (McGuire *et al*., 2014). Hence, *A. nigricollis* has a more conserved karyotype in relation to PAK suggesting that the fusion of GGA7 and GGA9 emerged after the divergence of these clades. In addition, this chromosomal rearrangement has not previously been observed in any bird group, according to data available in the Bird Chromosome Database (Degrandi *et al*., 2020b).

Although only eight species of hummingbirds have been karyotyped so far (three from this present study and five previously published), conventional chromosomal data is available also for six species of swifts, which belong to the family Apodidae, considered sister-group of Throchilidae: *Apus apus*: 2n=78, *Apus affinis affinis*: 2n=70, *Apus pacificus*: 2n=62, *Hirundapus caudacutus*: 2n= 64, *Streptoprocne zonaris*: 2n= 66, and *Streptoprocne biscutata*: 2n= 64 (XiaoZhuang and Qingwei 1989; Yadav et al., 1995; Ribeiro *et al*., 2003; Malinovskaya *et al*., 2018). Taking into account that hummingbirds and swifts share a common ancestor that must have existed 42 Ma (McGuire *et al*., 2014), a parsimonious scenario would point to an ancestor having a karyotype similar to the PAK. Additionally, despite being limited, these karyotypical data indicate that, while the hummingbirds have followed an evolutionary trajectory, maintaining a karyotype structure similar to the PAK (diploid numbers of 74-82 chromosomes), the chromosome complement of swifts have experienced a series of reductions, with diploid numbers decreasing to 62-78 chromosomes. However, more species need to be studied to determine which chromosomal events are acting on these species and to confirm whether this is an evolutionary trend or whether it is influenced by the low number of species that have been analyzed.

## Conclusions

The lack of cytogenetic data for hummingbirds is a major challenge for understanding karyotype evolution in this unique group of birds. The small number of species that have been karyotyped limits the scope of the analysis of chromosomal variation in this group. However, in the present study, chromosome painting demonstrated the occurrence of a fusion between homologues of GGA7 and GGA9 shared by *C*. *largipennis* and *H*. *chrysura*, reinforcing the molecular proposal that places these two species in the same clade, while *A*. *nigricollis* is found in a different clade. An important next step is to increase the number of species studied, including other clades of Trochilids, and also to use other chromosome painting probes from other species with more derives karyotypes, such as *Leucopternis albicollis*.
